# Mother-to-Child Transmission of Congenital Chagas Disease, Japan

**DOI:** 10.3201/eid2001.131071

**Published:** 2014-01

**Authors:** Kazuo Imai, Takuya Maeda, Yusuke Sayama, Kei Mikita, Yuji Fujikura, Kazuhisa Misawa, Morichika Nagumo, Osamu Iwata, Takeshi Ono, Ichiro Kurane, Yasushi Miyahira, Akihiko Kawana, Sachio Miura

**Affiliations:** National Defense Medical College, Saitama, Japan (K. Imai, T. Maeda, K. Mikita, Y. Fujikura, K. Misawa, M. Nagumo, T. Ono, Y. Miyahira, A. Kawana);; Japanese Red Cross Society, Tokyo, Japan (Y. Sayama, S. Miura);; Tokai University Oiso Hospital, Kanagawa, Japan (O. Iwata);; National Institute of Infectious Diseases, Tokyo (I. Kurane)

**Keywords:** congenital Chagas disease, mother-to-child transmission, Trypanosoma cruzi, parasites, megacolon, benznidazole, Japan

## Abstract

We report a patient with congenital Chagas disease in Japan. This report reemphasizes the role of neglected and emerging tropical diseases in the era of globalization. It also indicates the need for increased vigilance for detecting Chagas disease in non–disease-endemic countries.

Chagas disease, which is caused by the pathogenic protozoa *Trypanosoma cruzi*, was previously endemic only to Central and South America but is now estimated to affect up to 10 million persons worldwide (*1*). Recent unprecedented trends in globalization have been accompanied by the migration of ≈14 million persons from disease-endemic regions to North America, Europe, Japan, and Australia. Consequently, and as predicted, sporadic reports of patients with chronic Chagas disease have emerged, and documented cases have presumably been caused by chronically infected persons who migrated from disease-endemic countries (*2*). Despite the wide geographic spread of patients with Chagas disease, cases of congenital transmission in non–disease-endemic countries have been documented (Table 1) (*3*)

**Table 1:** Patients with congenital Chagas disease in non–disease-endemic countries*

Country	No. patients	Mother’s country of origin	Age, y, at time of diagnosis	Symptoms at birth
Sweden	1	Chile	5	Asymptomatic
Spain	7	Argentina (2), Bolivia (5)	At birth (5), 2 (1), after death (1)	Asymptomatic (5), symptomatic (2)
Switzerland	2	Bolivia (2)	At birth (2)	Asymptomatic (2)
United States	1	Bolivia	At birth	Symptomatic
Japan	1†	Bolivia	13	Asymptomatic

*Values in parentheses are no. patients. †Patient in this study.

It is estimated that ≈300,000 immigrants from Latin America, to which Chagas disease is endemic, are currently living in Japan and that ≈34,000 births from these immigrants have occurred in the past 10 years. However, vertical transmission of the disease in Japan has not been detected, probably because of the lack of screening programs for at-risk pregnant women and the disregard for the silent clinical manifestation of congenital Chagas disease.

The World Health Organization recommends that each country should strengthen its national and regional capacity to prevent and control congenital transmission of infectious pathogens while improving case management (*4*). We report a patient with congenital Chagas disease in Japan. We also highlight the need for increasing awareness of congenital transmission and urge establishment of an appropriate diagnostic and treatment system for Chagas disease in nonendemic countries.

## The Patient

In October 2012, a 13-year-old boy in Japan was admitted to the National Defense Medical College Hospital in Saitama, Japan, for chronic constipation. His parents and grandparents were Bolivian nationals of Japanese descent who had lived in Chagas disease–endemic areas in Bolivia until 1992. In 1999, the boy was delivered full-term after an uncomplicated pregnancy in Japan but had a low birthweight. He was in excellent health and showed no signs of disease until 2 years before his admission, when he began to report chronic constipation. At that time, he had a medical examination at a Catholic church because most hospitals in Japan could not make a definitive diagnosis of Chagas disease. He underwent serodiagnostic screening for *T. cruzi* infection. The boy and his mother were seropositive for *T. cruzi*.

After admission, he reported extreme constipation and explained that he defecated only once per week. Results of laboratory tests at admission, including those for serum brain natriuretic peptide, were generally within reference ranges. However, abdominal radiography showed major distension of the colon that extended 65 mm (Figure).

**Figure:**
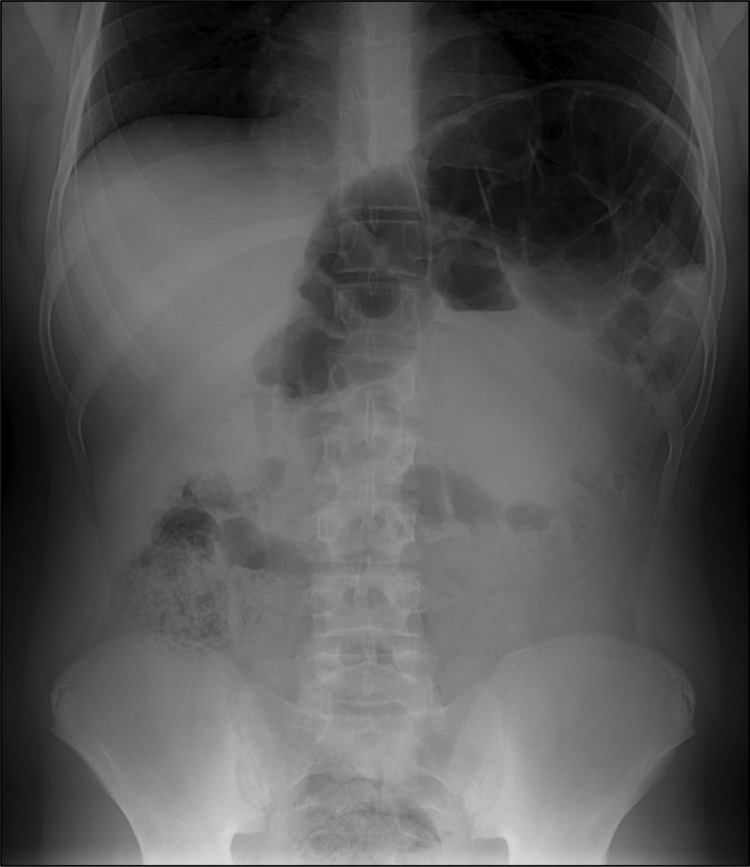
Abdominal radiograph of a 13-year-old boy with congenital Chagas disease, Japan, showing megacolon and marked dilatation at the splenic flexure.

The definitive diagnosis, including effectiveness of antiparasitic treatment, was confirmed by using serologic, genomic, and parasitologic methods (Table 2). An ELISA (ORTHO *T. cruzi* ELISA Test System; Ortho-Clinical Diagnostics, Raritan, NJ, USA) was performed according to the manufacturer’s protocol. A nested PCR that amplifies a DNA fragment of a repetitive TCZ sequence was performed as described (*5*). The parasite was also isolated by blood culture on Novy, McNeal, and Nicolle agar (*6*) and examined by light microscopy and real-time PCR. All tests showed positive results. It was later determined that the boy’s mother was also seropositive for Chagas disease. The boy was given a diagnosis of congenital Chagas disease accompanied by megacolon.

**Table 2:** Clinical course of a 13-year-old boy with congenital Chagas disease, Japan, after treatment with benznidazole

Characteristic	Before treatment	Days after starting treatment		
		30	60	180
Antibody titer*	160	160	160	160
Nested PCR result	+	+	–	–
Blood culture result	+	–	–	–

*Antibodies against *Trypanosoma cruzi*.

The patient was treated with oral benznidazole (5 mg/kg/d for 60 days) and showed no adverse effects. Parasitemia and DNA of *T. cruzi* in peripheral blood could not be detected by the end of treatment. To ensure successful treatment and cure, we intend to clinically follow up the patient for several decades until serologic results eventually become negative (*7*).

## Conclusions

Chagas disease is usually regarded as one of the most serious health problems in rural areas of Central and South America. However, recent successful vector control programs to reduce vector-borne transmission have dramatically changed the epidemiology of this disease (*8*). Mass migration of chronically infected and asymptomatic persons has caused globalization of Chagas disease, and has made nonvectorial infection, including vertical and blood-borne transmission, more of a threat to human communities than vectorial infection (*9*).

On the basis of local and limited serologic surveys, the presumptive number of chagasic patients living in Japan is currently 4,500, compared with >100,000 in the United States and >6,000 in Spain (*2**)*. Sporadic imported cases have been recognized and reported in Japan in the past decade, but the exact incidence is unknown. Most cases were diagnosed only after patients had critical complications, including severe cardiac involvement (*10*). It is also conceivable that chagasic patients with less severe cardiac symptoms or gastrointestinal involvement have sought treatment at local hospitals in Japan, where the potential for missing or misdiagnosing the disease would likely be high. The difficulty in making a correct diagnosis of Chagas disease is compounded in Japan by low awareness and recognition of the disease by medical staff; scarcity of epidemiologic or statistical data; and lack of diagnostic tools, resources, and facilities available to help with the differential diagnosis.

There is currently no laboratory test–based screening system for donated blood to detect Chagas disease in Japan. Instead, a questionnaire is used to determine if donors have any connections with disease-endemic regions. As of October 2012, to avoid transmission through transfusions, Japanese Red Cross Blood Centers no longer use donated blood for transfusions or producing blood products if the donor or donor’s mother has spent ≥4 weeks in Latin America. Therefore, before 2012, it is difficult to estimate how many contaminated blood donations were overlooked in Japan.

The estimated vertical transmission rate from an infected mother to her newborn is ≈5% in Bolivia (*11*). If one considers that 34,000 children were born to Latin American women during the past decade in Japan and that the seroprevalence of *T. cruzi* is estimated to be 1.8%, the number of infected newborns in the past decade is ≈30. However, there are no current screening programs for Chagas disease in Japan to detect chronically infected persons, including pregnant women and newborns.

The patient in this study had congenital chagasic infection, accompanied by advanced gastrointestinal complications. The delay in diagnosis for this patient case was caused by the absence of a screening program in Japan, a problem which also makes it impossible to determine the precise number of pregnant women and newborns with *T. cruzi* infection in this country. In Spain, the most affected country in Europe, a specific program was developed to focus on migrants from Latin American woman of childbearing age. Since its introduction, the program has contributed not only to the early diagnosis of Chagas disease but also to improvements in the quality of life and prognosis for patients (*12*).

Because the therapeutic efficacy of treatment, including benznidazole, for infection with *T. cruzi* is >90% in infants with congenitally transmitted Chagas disease if treated during the first year of life (*13*), it would be ideal for all pregnant women entering Japan from disease-endemic countries to be screened for the presence of serum antibody against *T. cruzi*. This report indicates the urgent need for implementing proper measures to prevent the vertical transmission of *T. cruzi* in non–Chagas disease–endemic countries, including Japan.
